# ﻿Distribution and morphology of the diatom genus *Olifantiella* Riaux-Gobin & Compère in Indonesian and Australian waters, including the description of *O.gondwanensis* sp. nov.

**DOI:** 10.3897/phytokeys.236.111109

**Published:** 2023-12-21

**Authors:** Mateusz Rybak, Sulastri Arsad, Catherine Riaux-Gobin, Oktiyas Muzaky Luthfi, Gustaaf Hallegraeff, Renata Ciaś, Agnieszka Kierzek, Andrzej Witkowski

**Affiliations:** 1 Department of Agroecology, Institute of Agricultural Sciences, Land Management and Environmental Protection, University of Rzeszów, Poland University of Rzeszów Rzeszów Poland; 2 Institute of Marine and Environmental Sciences, University of Szczecin, Szczecin, Poland University of Szczecin Szczecin Poland; 3 Faculty of Fisheries and Marine Science, University of Brawijaya, Brawijaya, East Java, Indonesia Universitas Brawijaya Brawijaya Indonesia; 4 Laboratoire d’Excellence ‘CORAIL’, University of Perpignan, Perpignan, France University of Perpignan Perpignan France; 5 CNRS-UPVD-EPHE, USR3278 CRIOBE, PSL Research University, Perpignan, France PSL Research University Perpignan France; 6 Institute of Marine and Antarctic Studies, University of Tasmania, Hobart, TAS, Australia University of Tasmania Hobart Australia

**Keywords:** Bacillariophyta, buciniportula, Diadesmidaceae, marine coasts, new combination, new species, taxonomy

## Abstract

Samples from coastal tropical waters of Central Sulawesi, Bangka Island and Bawean Island in Indonesia and from the Great Barrier Reef at Fitzroy Island in Queensland, Australia were analysed for species composition of diatom assemblages with a focus on *Olifantiella*. Whereas samples from Fitzroy Island littoral in Australia retrieved only one species of *Olifantiella*, in Poso Bay, Indonesia, we observed at least six species. All established taxa were documented with light (LM) and scanning electron microscope (SEM) and principal component analysis (PCA) analysis was used to compare the species, based on the basic valve parameters of length, width, length to width ratio and striae density. A new species of the genus *Olifantiella*, *O.gondwanensis* is described from Australia. In addition, we showed the distinct nature of O.pilosellavar.rhizophorae permitting to species status. Particular attention is placed on girdle bands in this genus.

## ﻿Introduction

*Olifantiella* Riaux-Gobin and Compère is a relatively recently described diatom genus consisting mainly of small, marine naviculoid diatoms (Riaux-Gobin and Compère 2009). To date, 12 taxa have been described within the genus, 11 at the species rank (Riaux-Gobin and Compère 2009; Riaux-Gobin and Al-Handal 2012; Van de Vijver et al. 2016; Kaleli et al. 2018; Van de Vijver et al. 2018; Jung and Park 2019; Wetzel and Ector 2020) and one variety (Riaux-Gobin 2015). The major characters of the genus are marginal elevated ridge, parallel to slightly radiate striae, composed of single transapically elongated macroareola with finely perforated hymenate occlusions which are not interrupted under the elevated ridge, simple raphe slit, several girdle bands and presence of a trumpet-like (‘olifant’) internal tubular process called buciniportula after which the genus is named (Riaux-Gobin and Al-Handal 2012). The exact function of the buciniportula is still unclear, but probably may have a function in excretion of metabolites (Riaux-Gobin 2015). Due to the small size of the cells and the mainly very dense striation, the identification of individual *Olifantiella* taxa is currently only possible using SEM. In addition, due to only single molecular sequences being available, genetic sequencing does not allow identification of most known taxa.

The genus *Olifantiella* exhibits high morphological similarity to other genera that possess a central isolated process, such as *Labellicula* Van De Vijver and Lange-Bertalot, *Luticola* D.G. Mann and *Luticolopsis* Levkov, Metzeltin & Pavlov. *Olifantiella* can be discriminated from *Labellicula*, based on differences in isolated pores (Riaux-Gobin and Compère 2009). Furthermore, both genera differ in the presence of a longitudinal channel in *Olifantiella*, but which is absent in *Labellicula* (Majewska et al. 2017). Additionally, the genus *Olifantiella* shows a small, heavily silicified nodule, placed between the proximal raphe endings (similar to *Neidium* Pfitzer), but which is absent in *Labellicula*. Moreover, the genus *Labellicula* shows curved T-shaped grooves on distal raphe endings, while in *Olifantiella*, the distal raphe endings are simple or only curved on one side. In contrast to *Olifantiella*, the monotypic genus *Luticolopsis* shows strongly apically twisted valves and striae composed of two rows of areolae (Levkov et al. 2013). The genus *Luticola* differs from *Olifantiella* mainly by its larger size, punctate striae and less tubular internal opening of the isolated process (Levkov et al. 2013). *Luticola* is also different in its ecological preferences, as the genus contains mainly freshwater and terrestrial species with only few taxa known from marine habitats (Levkov et al. 2013; Rybak et al. 2021). Han et al. (2018), based on molecular data of a Chinese *O.muscatinei* culture (strain DB21-1), confirmed that *OIifantiella* and *Luticola* are not only similar, but also closely related genera. Unfortunately, molecular data for *Labellicula* and *Luticolopsis* are currently not available and, hence, the degree of their evolutionary relationship with *Olifantiella* and *Luticola* remains unclear.

The genus seems to occur in various climatic zones of the world ocean, including temperate waters of the North Atlantic, South Atlantic and North West Pacific (Van de Vijver et al. 2016; Kaleli et al. 2018; Jung and Park 2019). However, most species have been described from the tropical Indian and Pacific Oceans. The first *Olifantiella* species was formally described from coral sands of Mascarenes in the Western Indian Ocean (Riaux-Gobin 2015), but retrospectively, it was realised that the first documented *Olifantiella* originated from the Gulf of Eilat in the Red Sea and originally characterised as *Naviculamuscatinei* Reimer and Lee = *Olifantiellamuscatinei* (syn. *O.pseudobiremis* Riaux-Gobin) present as an endosymbiont from the foraminiferan *Amphisteginalessonii* (Reimer and Lee 1988). Interestingly, *O.muscatinei* has also been isolated from foraminiferans of the Great Barrier Reef within which Fitzroy Island, the type habitat of our newly-described *Olifantiella* species, is located. A few *Olifantiella* species have been described from the tropical South Pacific (Riaux-Gobin 2015).

During our recent studies of the coastal diatom floras of Australia and Indonesia, a large number of *Olifantiella* were observed. Amongst them, an unknown species was recorded necessitating its description as a new species – *O.gondwanensis* M.Rybak, A.Witkowski & C.Riaux-Gobin, sp. nov. Additionally, updated information about the distribution and morphology of established taxa are given.

## ﻿Methods

Diatom samples were cleaned with 10% hydrochloric acid and washed thereafter with deionised water, followed by boiling with 30% hydrogen peroxide (H_2_O_2_) for a few hours and washed with deionised water. Cleaned diatom material was pipetted on to coverslips and dried and then mounted on glass slides using Naphrax mounting medium (Brunel Microscopes Ltd, Wiltshire, U.K.). Identification, counting and the measurements of diatom basic morphological features were performed under a Nikon ECLIPSE 80i light microscope (LM), equipped with a 100× Planapochromatic objective with differential interference contrast (DIC) for oil immersion (NA 1.4) and captured with a Nikon DS-Fi1c camera. For the observations in scanning electron microscope, the samples were applied to a polycarbonate membrane filter with a 3 μm mesh, attached to aluminium stubs and sputtered with 20 nm of gold using a turbo-pumped Quorum Q 150T ES coater. Diatoms were observed using a Hitachi SU 8010 SEM at University of Rzeszów, Poland. Diatom terminology follows Round et al. (1990) and Riaux-Gobin and Compère (2009).

The following samples were used in the present work:

- SZCZ 27565 – Bangka Island near the SE coast of Sumatra, 2°4'52.55"S, 105°8'14.65"E, 22 June 2022, sandy sediment.
- SZCZ 27652 – Bawean Island, North of Surabaya in the Central Sea of Java, 5°50′57.5′′S, 112°43′3.6′′E, 07 January 2021, the sample was taken from mangrove roots, temperature – 29 °C, salinity – 31‰.
- SZCZ 28814 – Indonesia, Sulawesi, Poso Regency, Tanjung Perak, 1°18′2.974′′S, 120°37′37.009′′E, 29 September 2022, the sample was collected during low tide as a scrape from exposed rock (depth ± 10–20 cm), temperature approximately above 30 °C, salinity – 28.5‰, pH – 7.76.
- SZCZ 28526 – Fitzroy Island, Australia, 16°55′38′′S, 149°59′24′′E, 15 August 2022, the sample was taken from green algae – *Chlorodesmis* sp. exposed on the Great Barrier Reef during the low tide.

To determine the similarity and/or dissimilarity of newly-described species (based on basic morphological features: length, width, length-to-width ratio and number of striae) with other members of the genus, Principal Component Analysis (PCA) was performed using Canoco 5.03 software (Ter Braak and Šmilauer 2012). Prior to analysis, diatom data were square-root transformed. Comparison was made, based on data from current observations and from the following publications: Riaux-Gobin and Compère (2009); Riaux-Gobin and Al-Handal (2012); Riaux-Gobin (2015); Kaleli et al. (2018) and Riaux-Gobin et al. (2019). Data for the following number of specimens were used for the analysis: *O.gondwanensis* sp. nov. (n = 42), *O.gorandiana* (n = 94), *O.infirmitata* (n = 7), *O.mascarenica* (n = 21), *O.muscatinei* (n = 15), O.cf.muscatinei (n = 25), *O.onnuria* (n = 7), *O.paucistriata* (n = 5), *O.pilosella* (n = 46), *O.rhizophorae* stat. nov. (n = 27), *O.rodriguensis* (n = 22), *O.seblae* (n = 33), *O.societatis* (n = 32) and *O.visurgis* (n = 26). In the case of *Olifantiellaonnuria*, data obtained from photographs included in their original description were used (Jung and Park 2019).

## ﻿Results

### ﻿Description of new species

**Class: Bacillariophyceae Haeckel, 1878**

**Subclass: Bacillariophycidae D.G.Mann in Round et al. (1990)**

**Order: Naviculales Bessey, 1907**

**Suborder: Neidiineae D.G.Mann in Round et al. (1990)**

**Family: Diadesmidaceae D.G.Mann in Round et al. (1990)**

**Genus: *Olifantiella* Riaux-Gobin & Compère**

#### ﻿ Olifantiella gondwanensis

Taxon classificationPlantaeNaviculalesNeidiineae

M.Rybak, A.Witkowski & C.Riaux-Gobin sp. nov.

42C7FECD-8491-570F-A53F-78BA9551158A

Fig. 2A–X

##### LM.

Valves small, elliptic to linear-lanceolate with broadly rounded apices, 6.3–13.7 μm long and 2.7–3.4 μm in width. Striae barely resolvable with LM, 26–31 in 10 μm, an isolated pore (buciniportula) visible near the central area.

##### SEM.

Striae composed of macroareolae, equidistant, with finely perforated hymen. At the junction of the valve face and the mantle, a ridge runs over the striae and opens to the exterior by round to oblong fenestrae, bordered by long thin spines – pili. Near the buciniportula, 1–2 shortened striae are present. Buciniportula located on opposite side of valves. Macroareolae on mantle the same as on valve and possessing long thin spines. Apical slits are few and small-sized without spines in their lumen. External raphe slits are straight and filiform. External proximal raphe endings slightly bent towards buciniportula bearing side, terminal raphe endings strongly hooked to the same side. Buciniportula opening sunken in the valve face connected with central raphe endings by small grooves. Small silica warts present externally along the raphe slits. Girdle bands numerous without ornamentation. Internally proximal raphe endings simple, terminal raphe endings forming small helictoglossae. Small siliceous warts present near the proximal raphe endings. Internally double buciniportula, slightly raised and closed. Longitudinal channels visible internally along the valve margin.

##### Type locality.

**Figure 1.**
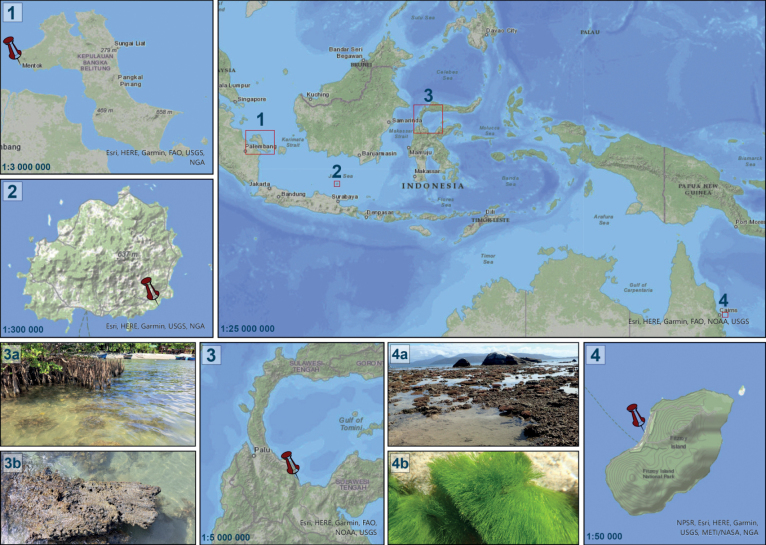
Locality of the sampling sites 1 Bangka Island 2 Bawean Island 3 Poso Pesisir, Sulawesi Island 3a, b view of the sampling site, photo. S. Arsad 4 type locality of *Olifantiellagondwanensis* sp. nov. in Fitzroy Island, Queensland, Australia 4a view of the type locality 4b green algae *Chlorodesmis* sp. from which sample was taken, photo. A. Witkowski.

Fitzroy Island, the Great Barrier Reef, Queensland, NE Australia, 16°55′38′′S ,149°59′24′′E (Fig. 1).

##### Type material.

**Figure 2.**
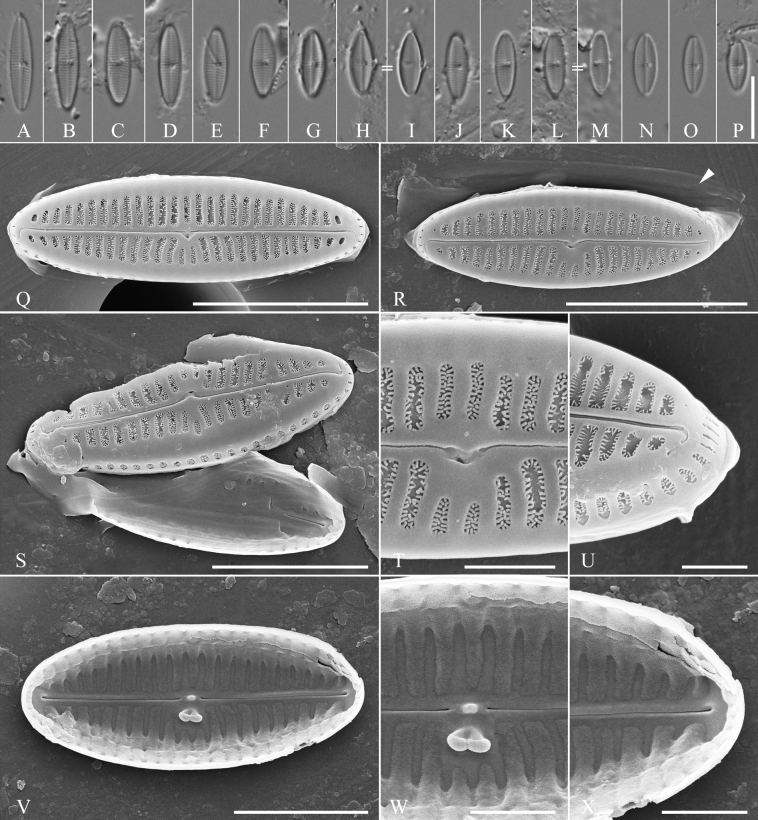
Holotype population of *Olifantiellagondwanensis* sp. nov. from Fitzroy Island **A–P** valves in size diminution series viewed by light microscopy **Q–X**SEM micrographs **Q, R** external view of the valve, white arrowhead indicates the girdle band **S** opened frustule **T** details of valve central part in external view **U** detail of valve apex in internal view **V** internal view of valve **W** details of valve central part in internal view **X** detail of valve apex in internal view. = indicates a valves of the same specimen. Scale bars: 10 µm (**A–P**); 5 µm (**Q, R**); 4 µm (**S**); 3 µm (**V**); 1 µm (**T, U, W, X**).

***Holotype***: Slide SZCZ 28526 and unmounted material with the same number in the collection of Andrzej Witkowski at the University of Szczecin. Holotype population is depicted in Fig. 2A–X.

***Isotype***: Slide number 2022/64 at the Diatom Collection at the University of Rzeszów.

##### Etymology.

The species epithet is derived from Gondwana, a super continent of which Australia was part of during the Paleozoic and Mesozoic Era.

##### Distribution.

So far observed only from the Australian type locality.

### ﻿Morphological characteristics of observed taxa

#### ﻿ Olifantiella gorandiana

Taxon classificationPlantaeNaviculalesNeidiineae

C.Riaux-Gobin

F3B4A1C0-2958-5D70-A0EB-D7C0650C854B

Fig. 3A–F

##### Description.

Valves elliptic to lanceolate with rounded to sub-rostrate apices, 4.8–6.8 μm long, 1.5–2.3 μm wide and with 58–67 striae in 10 μm. Buciniportula complex, internally with two short not raised tubular strctures flanked by two small satelites, external opening shifted near the valve margin. Buciniportula on both valves located on the same side of the frustule. Externally proximal raphe endings straight and slightly tear-drop-shaped, distal raphe endings with small, hooked grooves on site opposite to buciniportula. Internally proximal raphe endings rounded, distal raphe endings with small helictoglossa. Two types of girdle bands are present. The first one, wide with two rows of perforations (120–133 perforations in 10 μm) and second, thin without any perforations. Internally, this species shows a lack of longitudinal channel.

**Figure 3.**
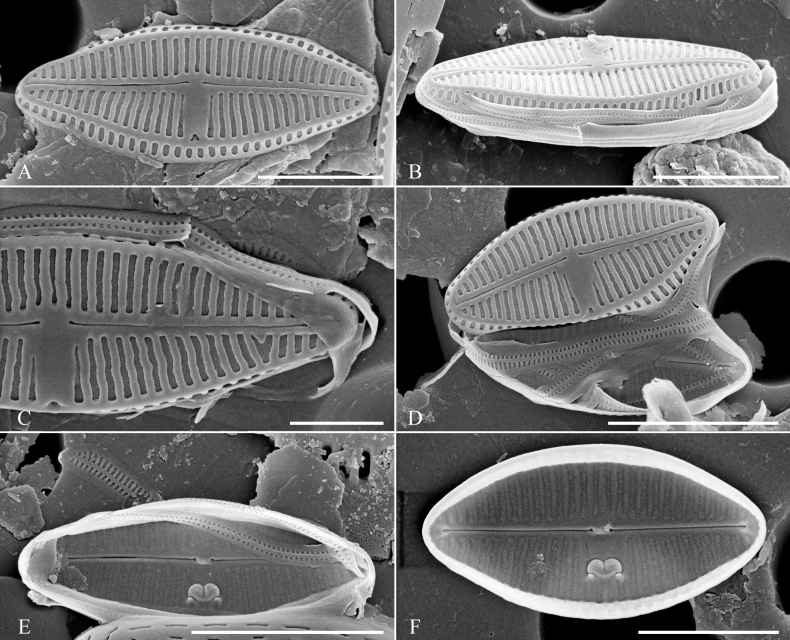
SEM documentation of *Olifantiellagorandiana* from Sulawesi Island coast (sample SZCZ 28526). Scale bars: 3 µm (**B, D**); 2 µm (**A, E, F**); 1 µm (**C**).

##### Distribution.

Newly observed from coastal waters of Sulawesi Island (sample SZCZ 28814). Originally reported from Rodrigues Island and also observed in French Polynesia, Western Indian Ocean (Riaux-Gobin 2015; Riaux-Gobin et al. 2019), Galapagos Island and West Coast of United States (Witkowski – unpublished data).

##### Comments.

All observed specimens represent the morphotype ‘b’ following Riaux-Gobin et al. (2019).

#### ﻿ Olifantiella mascarenica

Taxon classificationPlantaeNaviculalesNeidiineae

C.Riaux-Gobin & Compère

4AE9ABF8-5D5B-5675-82AC-FE0266F31DCD

Fig. 4G–J

##### Description.

Valves linear with capitate apices, 6.4–11.8 μm in length, 2.0–2.3 μm in width and with 41–55 striae in 10 μm. Buciniportula complex, internally with a single raised tubular process. A single shortened stria is present near the valve margin on the side of the buciniportula. Apical slits narrower than macroareola. Girdle bands with two rows of perforations (ca. 110 pores in 10 μm). Externally proximal raphe endings straight and tear-drop-shaped, distal raphe endings slightly bent towards buciniportula site. Narrow longitudinal channels visible internally along the valve margin.

**Figure 4.**
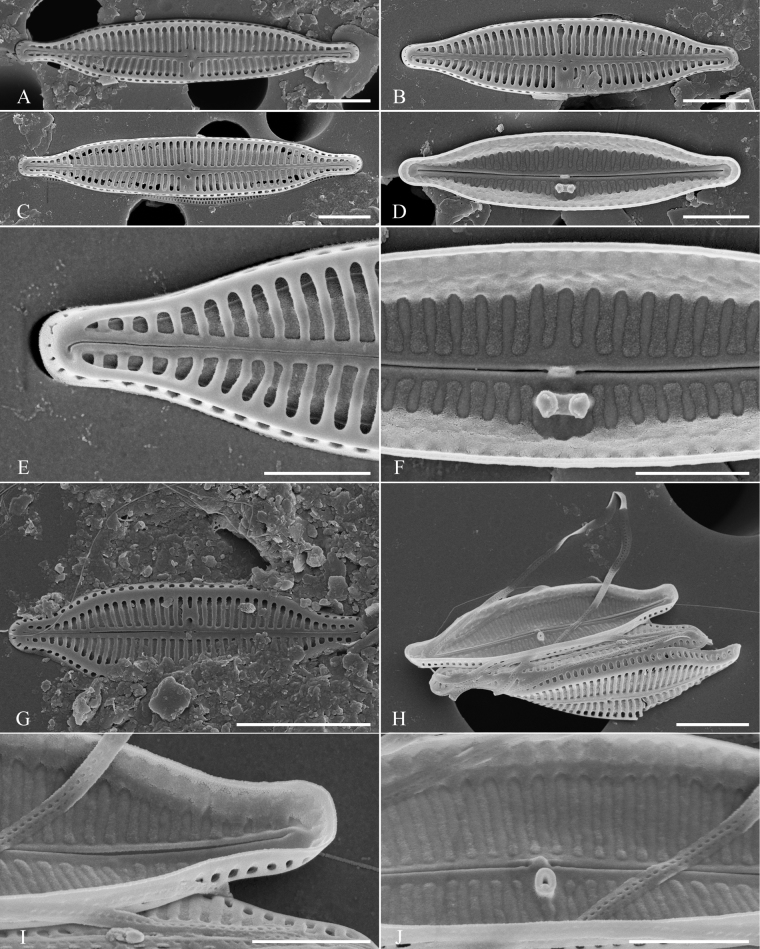
SEM documentation of *Olifantiellarodriguensis* (**A–F**) and *Olifantiellamascarenica* (**G–J**) **B–J** population from Sulawesi Island coast (sample SZCZ 28814) **A** specimen from Bangka Island (sample SZCZ 27565). Scale bars: 3 µm (**E**); 2 µm (**A–D, H**); 1 µm (**E, F, I, J**).

##### Distribution.

Newly observed from coastal waters of Sulawesi and Bawean Islands (samples SZCZ 28814 and SZCZ 27652). Originally reported from Rodrigues Island, also observed in Moorea Island (Riaux-Gobin 2015) and coast of Turkey (Kaleli 2022).

#### ﻿ Olifantiella paucistriata

Taxon classificationPlantaeNaviculalesNeidiineae

C.Riaux-Gobin

5D78AAA1-FC82-5008-B6AA-AAEA79CDD151

Fig. 5A

##### Description.

Valves small, elliptic with rounded apices, 6.1 μm in length, 2.7 μm in width and 31 striae in 10 μm. Striae composed of macroareolae, equidistant, with finely perforated hymen. External buciniportula opening sunken in the valve face connected with central raphe endings by small grooves. On the buciniportula-bearing side, 1–2 shortened striae are present. External proximal raphe endings straight with small grooves, terminal raphe endings strongly hooked towards buciniportula side.

**Figure 5.**
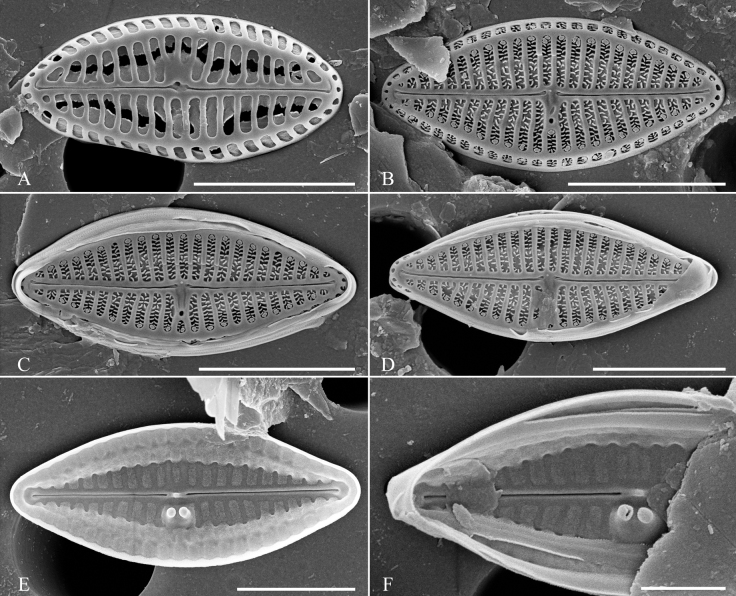
SEM documentation of *Olifantiellapaucistriata* (**A**) and *Olifantiellapilosella* (**B–F**) from from Sulawesi Island coast (sample SZCZ 28814). Scale bars: 3 µm (**A–D**); 2 µm (**E**); 1 µm (**F**).

##### Distribution.

Newly observed from coastal waters of Sulawesi Island (sample SZCZ 28814). Originally reported from Rodrigues Island (Riaux-Gobin and Al-Handal 2012).

#### ﻿ Olifantiella pilosella

Taxon classificationPlantaeNaviculalesNeidiineae

C.Riaux-Gobin

D1144233-7C85-51AE-9343-B1973838F47E

Fig. 5B–F

##### Description.

Valves elliptic to linear-elliptic with rounded apices, 5.4–8.2 μm in length, 2.3–2.8 μm in width and with 38–43 striae in 10 μm. Macroareolae, both on valve face and valve mantle bordered by thin siliceous spines (pili) and small siliceous plates (flabella). Between buciniportula opening and valve margin, one shortened stria is present. Apical slits small, without silica projections. Buciniportula complex, internally with two short raised tubular processes. Externally, both proximal and distal raphe endings are tear-drop-shaped and slightly bent opposite to the buciniportula opening with elongate grooves on buciniportula site. Internally raphe endings are straight and simple, forming small helictoglossae at distal endings. Girdle bands numerous with barely visible perforations. Internally, small silica warts between proximal raphe endings are present. Broad longitudinal channels visible internally along the valve margin.

##### Distribution.

Newly observed from coastal waters of Sulawesi Island (sample SZCZ 28814). Originally described from Rodrigues Island and afterwards observed in Moorea Island (Riaux-Gobin and Al-Handal 2012; Riaux-Gobin 2015). Additionally observed in Mariana Islands, Guam (Lobban et al. 2012).

#### ﻿ Olifantiella rodriguensis

Taxon classificationPlantaeNaviculalesNeidiineae

C.Riaux-Gobin

4BF27E63-D307-5593-8947-E7B082DC3FB4

Fig. 4A–F

##### Description.

Valves linear with capitate apices, 9.0–12.8 μm in length, 2.0–2.3 μm in width and with 41–43 striae in 10 μm. Buciniportula complex, internally with two short raised tubular processes. Two shortened striae are present near the valve margin on the side of the buciniportula. Apical slits narrower than macroareola. Girdle bands with two rows of perforations (ca. 100 perforations in 10 μm). Externally proximal raphe endings straight and tear-drop-shaped, distal raphe endings slightly bent towards buciniportula location. Broad longitudinal channels visible internally along the valve margin.

##### Distribution.

Newly observed from coastal waters of Sulawesi and Bangka Island (samples SZCZ 28814 and SZCZ 27565). Originally reported from Rodrigues Island and later observed from Galapagos Islands (Riaux-Gobin and Al-Handal 2012; Riaux-Gobin 2015; Witkowski et al. – unpublished data).

#### ﻿ Olifantiella societatis

Taxon classificationPlantaeNaviculalesNeidiineae

C.Riaux-Gobin

323B5E74-93B8-5661-B01B-5A3B7E3D3D56

Fig. 6A–F

##### Description.

**Figure 6.**
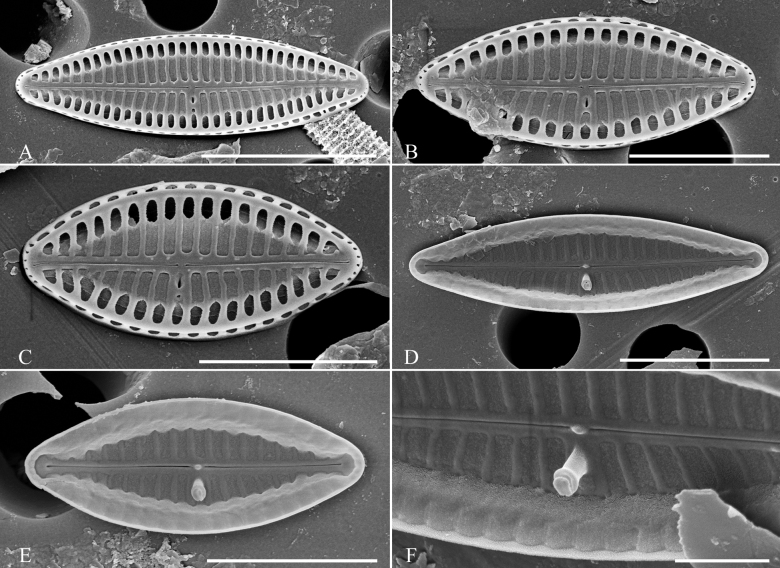
SEM documentation of *Olifantiellasocietatis* from Sulawesi Island coast (sample SZCZ 28814). Scale bars: 5 µm (**A**); 4 µm (**D**); 3 µm (**B, C, E**); 1 µm (**F**).

Valves elliptic to linear-elliptic with rounded apices, 3.9–10.3 μm in length, 2.2–2.8 μm in width and with 31–34 striae in 10 μm. Macroareolae are present at valve face and valve mantle. External buciniportula opening elongate, internally in form of singular tubular process, with a “*Nepenthes*-like” plug (Fig. 6F) as in *O.mascarenica* (Riaux-Gobin and Compère 2009; Riaux-Gobin and Al-Handal 2012). A short macroareola (not observed in *O.societatis* type) is present at the prolongation of the buciniportula aperture. At the junction of the valve face and the mantle, a ridge runs over the striae and opens to the exterior by round to oblong fenestrae. Externally, both proximal and distal raphe endings are straight and tear-drop-shaped. Internally raphe endings are straight and simple, forming small helictoglossae at distal endings. Small silica warts are present externally along raphe slits. Internally two broad longitudinal channels and small silica warts between proximal raphe endings are present.

##### Distribution.

Newly observed from the coast of Sulawesi Island (sample SZCZ 28814), Bangka Island (SZCZ 27565) and Bawean Island (SZCZ 27652). Originally reported from Moorea Island (Riaux-Gobin 2015).

### ﻿Comparison of basic morphological characters of known taxa

Principal component analysis (PCA) revealed considerable variability in the morphological characters of the *Olifantiella* species considered. The gradient length in analysis was 0.4. The first ordination axis eigenvalue was 0.95 and the second 0.04 (Fig. 6). The newly-described species clearly distinguished itself from the others. Additionally, based on the results of the PCA analysis showing the separation of the *O.pilosella* group from the group of its variety, we propose the following taxonomic change:

#### ﻿ Olifantiella rhizophorae

Taxon classificationPlantaeNaviculalesNeidiineae

(C.Riaux-Gobin) M.Rybak, A.Witkowski & C.Riaux-Gobin stat. nov.

D18DB672-5066-5EE8-907B-0CE5293D3910

Olifantiella pilosella var. rhizophorae C.Riaux-Gobin 2015: Botanica Marina 58(4): 255–258, figs 9–16. Basionym.

## ﻿Discussion

Despite its small size and more than a dozen of species discriminated, the genus *Olifantiella* presents great morphological diversity. This manifests itself in the shape of the valves, the number and structure of the internal buciniportula openings, the structure of the areolae and in the details of the distal and proximal raphe endings.

**Figure 7.**
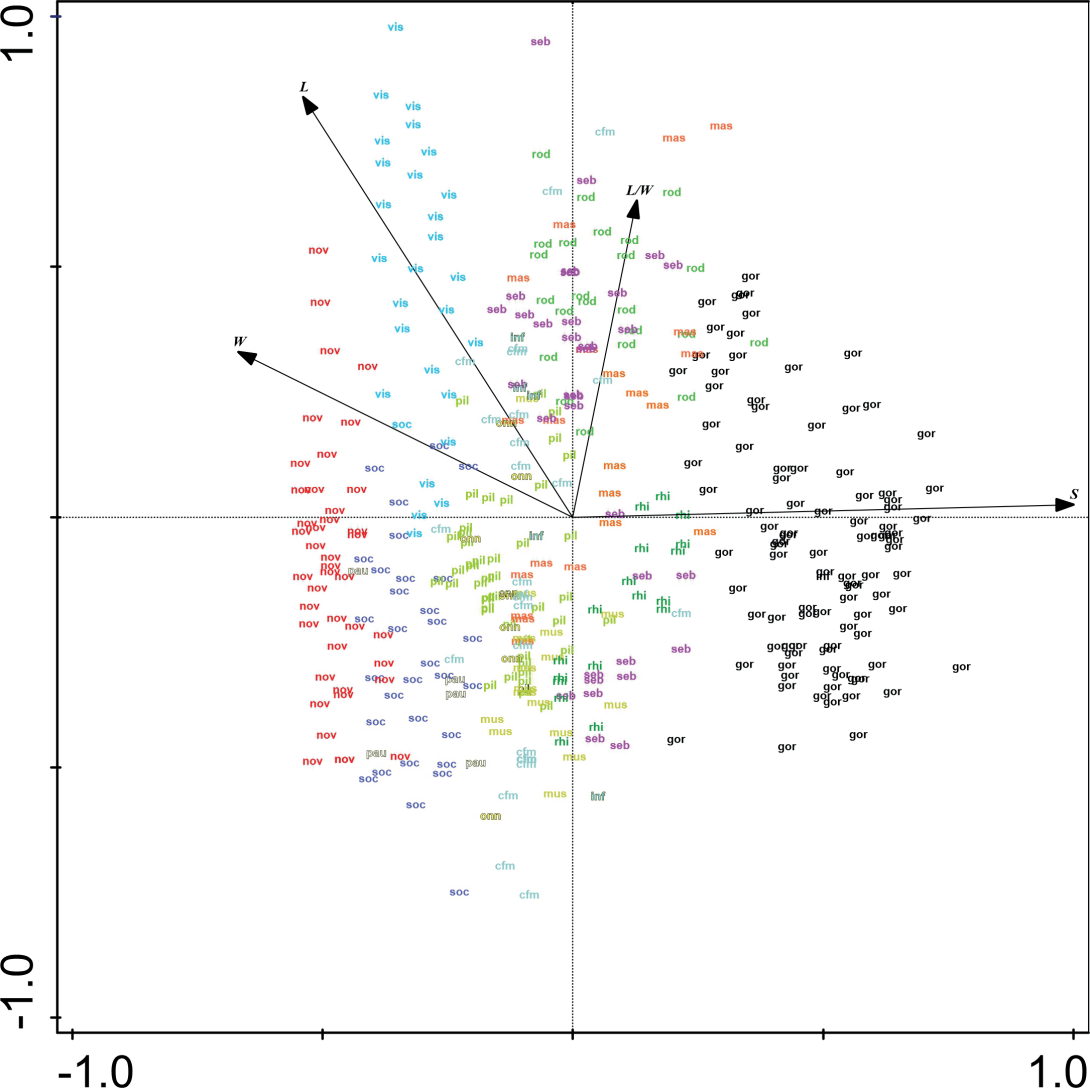
PCA ordination of all currently known *Olifantiella* taxa, based on their basic morphological features **L** length **W** width **L/W** length/width ratio **S** stria density **nov***O.gondwanensis* sp. nov. **rhi***O.rhizophorae* stat. nov. **vis***O.visurgis***gor***O.gorandiana***mas***O.mascarenica***cfm**O.cf.mascarenica**inf***O.infirmitata***seb***O.seblae***pil***O.pilosella***rod***O.rodriguensis***soc***O.societatis***pau***O.paucistriata***mus***O.muscatinei***onn***O.onnuria*.

Amongst known *Olifantiella* taxa, the newly-described *O.gondwanensis* sp. nov. is characterised by the lowest striae density (Fig. 7), which makes it the only species that can be easily identified using light microscopy. The greatest similarity to the newly-described species is shown by *O.pilosella*, *O.rhizophorae*, *O.paucistriata*, *O.gorandiana* and *O.muscatinei* (Reimer and J.J.Lee) Van de Vijver, Ector and C.E.Wetzel. Interestingly, *O.muscatinei* has also been isolated from foraminiferans of the Great Barrier Reef within which Fitzroy Island, the type habitat of our newly-described *Olifantiella* species, is located.

*Olifantiellapilosella* like the newly-described species, has areolae bearing long thin spines (pili), but in contrast to *O.gondwanensis* sp. nov., it has additional siliceous plates (flabella) in the areolae lumen (Riaux-Gobin and Al-Handal 2012; Riaux-Gobin 2015) which are absent in our newly-described species (Fig. 2Q–U). Additionally, both species can be separated, based on the external opening of the buciniportulae, which are positioned midway between the valve centre and valve margin in *O.pilosella*, but positioned in a small depression close to the proximal raphe endings in *O.gondwanensis* sp. nov. Internal views also show that *O.pilosella* possesses wider longitudinal channels when compared to the whole valve than *O.gondwanensis* sp. nov. (Fig. 2V–X vs. Fig. 5E, F). Another feature that allows the two species to be distinguished is the more elliptical shape of the vales in the newly-described species when compared to *O.pilosella*.

In addition, due to the elliptical shape of the valves, it is possible to distinguish *O.gondwanensis* sp. nov from the similar *O.rhizophorae* stat. nov. The latter species is also characterised by the simpler structure of areolae that show only short warts on the margin, instead of long and thin pili. Moreover, the external opening of buciniportula is shifted near the valve margin in *O.rhizophorae* stat. nov. (Riaux-Gobin 2015), but not positioned almost in the valve centre like in *O.gondwanensis* sp. nov. Additionally, *O.rhizophorae* stat. nov. possesses simpler raphe branches, which have both proximal and distal endings gently bent to the side opposite to the buciniportula (Riaux-Gobin 2015). This feature distinguishes this species from both *O.gondwanensis* sp. nov. and *O.pilosella* which have shallow depressions at both raphe endings (Riaux-Gobin and Al-Handal 2012; Riaux-Gobin 2015, Fig. 5B–D)

*Olifantiellapaucistriata* is the least known member of the genus, with fewer than 10 individuals of this species documented so far (Riaux-Gobin and Al-Handal 2012). Despite that fact and despite the similar shape of the valve to *O.gondwanensis* sp. nov., both species can be easily discriminated. The main distinguishing characters are the striae density (higher in *O.paucistriata*, see Table 1) and presence of the pili in the areolae lumen in *O.gondwanensis* sp. nov. However, similar structures of the raphe, location of the external opening of the buciniportula near the valve centre and the presence of small warts in the areolae lumen in *O.paucistriata* indicate its close relationship to *O.gondwanensis* sp. nov. This requires further research supported by better documentation for the first of these species.

**Table 1.** Comparison of *Olifantiellagondwanensis* sp. nov. with most similar *Olifantiella* taxa based on valve morphology.

	*O.gondwanensis* sp. nov.	*O.gorandiana*	*O.muscatinei*		*O.pilosella*	*O.rhizophorae* stat. nov.	*O.paucistriata*
**shape**	linear-lanceolate	elliptical, slightly elongated apices	elliptic-lanceolate	variable, depending on size, linear-lanceolate, lanceolate or elliptic-lanceolate	elliptical to oblong-elliptical, slightly elongate apices	elliptical with slightly acuminate apices	naviculoid
**length (µm)**	6.3–13.7	4.0–9.0	2.5–6.0	3.6–11.7	7.4–11	5.4–7.8	6.0–8.0
**width (µm)**	2.7–3.4	0.7–3.0	2.0–2.5	1.3–3.4	1.8–3	1.4–2.3	1.8–2.0
**striae (in 10 µm)**	26–31	52–70	28–30	37–52	30–41	40–47	31–37
**macroareolae structure**	occluded by finely perforated hymen, bordered by long thin spines	occluded by finely perforated hymen	no data	occluded by finely perforated hymen	occluded by finely perforated hymen, bordered by long thin spines	occluded by finely perforated hymen	occluded by finely perforated hymen
**external process opening**	Round to elliptical, deeply depressed	trapezoidal, deeper split into four sectors, close to margin	no data	single foramen-like opening	tear-like, in mid-stria	round, near the margin	round, small-sized, close to central area
**external central raphe endings**	expanded and deflected to the external opening of buciniportula	inflated, slightly deflected away from process opening	no data	expanded and deflected away from the external opening of buciniportula	slightly inflated, deflected away from the foramen	deflected away from foramen	slightly inflated, not deflected
**external terminal raphe endings**	strongly deflected to buciniportula opening side	simple, straight	no data	expanded and in the same direction as central raphe endings	simple, deflected opposite foramen	simple, deflected opposite to foramen	simple, slightly deflected opposite foramen
**buciniportula**	double, slightly raised, closed	multiple, flattened, (not erected), with two satellites	no data	double, each covered by finely perforated, domed thickening	double, raised, closed	double, raised, closed	no data
**modified or shortened striae**	2	4	no data	2–3	1	1	2
**Type locality**	Australia, Fitzroy Island	Western Indian Ocean (Rodrigues Island)	Gulf of Eilat (Israel)		Western Indian Ocean (Rodrigues Island)	Moorea Island (South Pacific)	Western Indian Ocean (Rodrigues Island)
**source**	this study	Riaux-Gobin and Al-Handal 2012; Riaux-Gobin et al. 2019	Reimer and Lee 1988	Riaux-Gobin and Al-Handal 2012; Jung and Park 2019	Riaux-Gobin and Al-Handal 2012; Riaux-Gobin 2015	Riaux-Gobin 2015	Riaux-Gobin and Al-Handal 2012

*Olifantiellamuscatinei* (syn. *O.pseudobiremis* Riaux-Gobin) not only has a valve similar in shape to the newly-described species, but also overlapping size dimensions including length, width and striae density (see Table 1). Nevertheless, the distinction of both species is possible on the basis of morphological features of its valves and girdle bands. *O.muscatinei* is the only known member of the genus showing well-developed external perforated areola occlusions, both on the valve face and vale mantle (Riaux-Gobin and Al-Handal 2012; Jung and Park 2019). Additionally, the external opening of the buciniportula is shifted more towards the edge of the valve than with *O.gondwanensis* sp. nov. An additional difference between the two species is their girdle bands. They are hyaline in *O.gondwanensis* sp. nov. (Fig. 1R, S), while *O.pseudobiremis* has girdle bands with double rows of pores (Riaux-Gobin and Al-Handal 2012; Jung and Park 2019).

Of the five described morphotypes of *O.gorandiana* (Riaux-Gobin et al. 2019), the greatest similarity to *O.gondwanensis* sp. nov. is shown by the morphotypes ‘a’ and ‘b’. However, based on morphological characterss of the valves, both species are easily separated. Regardless of the morphotype, *O.gorandiana* has definitely denser striae (see Table 1), composed of simple areolae without any silica projections (Fig. 3A–D; Riaux-Gobin and Al-Handal 2012; Riaux-Gobin et al. 2019). In addition, the external opening of the buciniportula is strongly shifted to the edge of the valve face in *O.gorandiana* and not centrally positioned like in the case of *O.gondwanensis* sp. nov.

*Olifantiellagorandiana* is the only species in the genus within which several morphotypes have been distinguished, based on the shape of the valves. Based on the observation of a population developing epizoic on sea turtle (*Cheloniamydas* Linnaeus, 1758), five morphotypes have been distinguished (Riaux-Gobin et al. 2019). In the population documented in this work, all observed individuals represented the ‘b’ morphotype. However, the observed population showed a polymorphism in the construction of girdle bands. Most of them showed two rows of puncta (Fig. 3B–D), while some specimens also possessed plain girdle bands without any pores (Fig. 3C). The presence of two different types of girdle bands in a single species, even in single specimens, has not been observed so far in the genus *Olifantiella*. Most species within the genus have girdle bands with clearly visible two rows of pores, which were originally considered to be a characteristic feature of the genus (Riaux-Gobin and Compère 2009). However, later described species showed some plasticity in this character. For example, the here-described *O.gondwanensis* sp. nov. and *O.rhizophorae* stat. nov. have girdle bands without pores (Fig. 2R, S; Riaux-Gobin 2015). Additionally, *O.pilosella* also shows girdle bands which are not typically with two rows of areolae, but possess small and poorly visible pores (Fig. 4C). It seems that the morphology of the girdle bands is not a good criterion for defining members of this genus.

Studies focusing on *Olifantiella* have been conducted in Mascarenes (La Réunion, Rodrigues), Tahiti and Moorea Islands and coastal waters of the Mediterranean, Red, Baltic and North Seas. Until now, the occurrence of this genus has not been observed in the coastal waters of south-east Asia as well as in Australia, except the Great Barrier Reef area. Despite the great morphological diversity of representatives of the genus *Olifantiella*, only a few species are known, which may be due to their small size and, thus, difficulties in their observation and even detection in the studied materials. Future studies of coastal waters may result in descriptions of further members of this elusive genus. Additionally, further research (including molecular methods) is required to determine the relationships within the genus and with the other isolated pore-bearing members of family Diadesmidaceae, especially with the morphologically similar genus *Labellicula*.

## Supplementary Material

XML Treatment for Olifantiella gondwanensis

XML Treatment for Olifantiella gorandiana

XML Treatment for Olifantiella mascarenica

XML Treatment for Olifantiella paucistriata

XML Treatment for Olifantiella pilosella

XML Treatment for Olifantiella rodriguensis

XML Treatment for Olifantiella societatis

XML Treatment for Olifantiella rhizophorae

## ﻿Additional information

### Author ORCIDs

Mateusz Rybak http://orcid.org/0000-0001-8998-9537

Sulastri Arsad https://orcid.org/0000-0002-7322-7834

Catherine Riaux-Gobin https://orcid.org/0000-0002-6128-8947

Oktiyas Muzaky Luthfi https://orcid.org/0000-0002-9550-9381

Gustaaf Hallegraeff https://orcid.org/0000-0001-8464-7343

Renata Ciaś https://orcid.org/0009-0000-9124-3734

Agnieszka Kierzek https://orcid.org/0000-0001-8023-5114

Andrzej Witkowski http://orcid.org/0000-0003-1714-218X

### Data availability

All of the data that support the findings of this study are available in the main text.
